# Formulation and Evaluation of Xanthan Gum Microspheres for the Sustained Release of Metformin Hydrochloride

**DOI:** 10.3390/mi14030609

**Published:** 2023-03-06

**Authors:** Madiha Melha Yahoum, Selma Toumi, Hichem Tahraoui, Sonia Lefnaoui, Mohammed Kebir, Abdeltif Amrane, Aymen Amin Assadi, Jie Zhang, Lotfi Mouni

**Affiliations:** 1 Materials and Environmental Laboratory (LME), University of Medea, Nouveau Pole Urbain, Medea 26000, Algeria; 2 Faculty of Sciences, University of Medea, Nouveau Pole Urbain, Medea 26000, Algeria; 3 Laboratoire de Génie des Procédés Chimiques, Department of Process Engineering, University of Ferhat Abbas, Sétif 19000, Algeria; 4 Laboratory of Biomaterials and Transport Phenomena (LBMTP), University Yahia Fares, Médéa 26000, Algeria; 5 Laboratory of Experimental Biology and Pharmacolgy, University of Medea, Nouveau Pole Urbain, Medea 26000, Algeria; 6 Research Unit on Analysis and Tecshnological Development in Environment (URADTE-CRAPC), MB 384, Tipaza 42000, Algeria; 7 National Center for Scientific Research (CNRS), National School of Chemistry of Rennes, University of Rennes, ISCR—UMR6226, F-35000 Rennes, France; 8 College of Engineering, Imam Mohammad Ibn Saud Islamic University, IMSIU, Riyadh 11432, Saudi Arabia; 9 School of Engineering, Merz Court, Newcastle University, Newcastle upon Tyne NE1 7RU, UK; 10 Laboratory of Management and Valorization of Natural Resources and Quality Assurance, SNVST Faculty, Akli Mohand Oulhadj University, Bouira 10000, Algeria

**Keywords:** xanthan gum, metformin chloride, encapsulation, gelling, extended release

## Abstract

This work aimed to formulate xanthan gum microspheres for the encapsulation of metformin hydrochloride, according to the process of ionotropic gelation. The obtained microparticles, based on various fractions of xanthan gum (0.5–1.25), were subjected to different physico-chemical tests and a drug release study. Microspheres with an average size varying between 110.96 μm and 208.27 μm were obtained. Encapsulation efficiency reached 93.11% at a 1.25% biopolymer concentration. The swelling study showed a swelling rate reaching 29.8% in the gastric medium (pH 1.2) and 360% in the intestinal medium (pH 6.8). The drug release studies showed complete metformin hydrochloride release from the beads, especially those prepared from xanthan gum at the concentration of 1.25%, in intestinal medium at 90.00% after 6 h. However, limited and insignificant drug release was observed within the gastric medium (32.50%). The dissolution profiles showed sustained release kinetics.

## 1. Introduction

Improving the quality of drug treatments has always been one of the major concerns of humans. It is within this framework that technical progress is generally dependent on the development of much more efficient systems based on new materials with improved properties and better adapted to current health requirements. Modified-release forms such as hydrophilic matrices [1,2] or microparticles such as microspheres and microcapsules fit into this perspective [3].

Microencapsulation is a process by which it is possible to produce individualized microparticles using a polymeric material intended to contain an active substance in an amount of 5% to 90%. This technique is applied to several fields, such as food, pharmacy, cosmetics, or other fields including catalysis. In the pharmaceutical field, encapsulation is used for different purposes, namely the immobilization and the protection of the active ingredient, masking some of its undesirable organoleptic proprieties, such as odor, or even making it possible to control and trigger the release of drugs [4,5].

Microparticles are divided into two distinct types: microcapsules and microparospheres. Microcapsules are reservoir particles made up of a solid or (a more or less) viscous liquid core of active substance, surrounded by a continuous solid shell of coating material. The microspheres are particles made up of a continuous macromolecular or lipid network forming a matrix in which the active ingredient is finely dispersed, in the form of molecules of fine solid particles or even droplets of solutions [3].

Polysaccharides represent the largest category of materials used as encapsulating agents, due to their multiple advantages, particularly their biocompatibility and biodegradability, as well as their low toxicity and gelling capacity. Among these biopolymers, alginate, pectin or chitosan, and xanthan gum, are largely used for the encapsulation of active substances such as drugs and essential oils, as well as viable cells [6,7,8]. In the present work, the choice was directed towards xanthan gum due to its valuable proprieties and biosafety, and also for its rheological characteristics and high viscosity, which make it stand out from the rest of the biopolymers. Furthermore, xanthan gum is non-toxic to health and to the environment, having a production route considered to be sustainable [9]. Xanthan gum, due to its specific proprieties, is currently used in drug delivery systems for sustained-release activity. Formulations based on xanthan gum offer an effective sustained release due to the more branched polysaccharide structure than other biopolymers, such as alginate, pectin or chitosan, due to which the drug is entrapped, hence decreasing its diffusion [9].

Xanthan gum (XG) is a branched anionic heteropolysaccharide produced by a Gram (-) bacterium, *Xanthomonas compestris*. The primary structure of xanthan gum consists of a chain of pentasaccharide units. Each unit contains D-glucose, D-mannose and D-glucuronate in a 2:2:1 molar ratio, respectively. In an aqueous solution, xanthan gum exhibits an ordered helical conformation as a quintuple helix. This anionic polysaccharide is completely soluble in hot or cold water and hydrates rapidly once dispersed, facilitating water retention and producing highly viscous solutions even at low concentrations. Moreover, xanthan is characterized by its high thermal stability and exceptional rheological properties. Indeed, this biopolymer exhibits excellent thermal stability, where its solutions retain uniform viscosities over a wide range of temperatures [9]. Furthermore, it was recently reported that xanthan gum has a potential blood-sugar-lowering and -stabilizing effect. This last particular property makes xanthan gum an excellent candidate for the administration of active antidiabetic substances [10,11].

Metformin hydrochloride (MTH) is an antidiabetic active ingredient belonging to the biguanide family [12], administered for the treatment of type II diabetes as a regulator of blood sugar levels through an increase in insulin action and the inhibition of gluconeogenesis [13]. Metformin improves hepatic insulin sensitivity and is therefore recommended in the treatment of polycystic ovary syndrome in obese patients with insulin resistance [14]. Some researchers reported the potential anticancer activity of metformin against breast cancer [15].

Metformin is characterized by its low oral bioavailability and short half-life of around 1.5 to 3 h [16], requiring several administrations per day (500 mg or 850 mg once to twice daily), hence negatively influencing the good compliance with the treatment and therefore its effectiveness, and it also causes gastrointestinal discomfort such as diarrhea. Additionally, when metformin hydrochloride is administered orally, it undergoes alterations as it passes through the digestive tract, where the pH is very acidic, in addition to the presence of enzymes. These major drawbacks make metformin a good candidate for sustained release by using a suitable dosage form such as polymeric microparticles or microcapsules. In addition to the control of the drug release, these systems offer the more effective protection of active ingredients against degradation (pH, enzymes), thus increasing the bioavailability, in addition to better patient compliance and an increase in comfort and safety via a greater reduction in side effects [17].

Several studies have reported the use of polymeric microparticle encapsulation systems for the sustained release of MTH using diverse types of polymers and preparation techniques. MTH mucoadhesive microcapsules based on sodium alginate and gum karaya were developed by Kumar et al. [18], using emulsification ionotropic gelation. Another study was conducted by Nath et al. [19] on the development of floating microcapsules for metformin hydrochloride based on Eudragit RL 100 and cellulose acetate butyrate by the nonaqueous emulsion–solvent evaporation method. Silva et al. [20] used poly(L-lactic acid) and carboxymethyl cellulose microparticles produced by the double emulsion–solvent evaporation technique for MTH sustained release. Furthermore, Choudhury et al. [21] prepared cellulose acetate microspheres by emulsion–solvent evaporation for metformin hydrochloride’s gastric retention. Pectin microspheres were developed by Banerjee et al. [22], using different ratios of the drug pectin, and also with different polymers, namely ethyl cellulose, hydroxypropylcellulose (HPMC) and Acrycoat S100. Bioadhesive chitosan microparticles for the oromucosal drug administration of MTH were prepared by Madsen et al. [23], using the spray-drying method. Hariyadi et al. [24] resorted to an ionotropic gelation technique for the preparation of MTH-loaded alginate microspheres. *Cydonia oblonga* mucilage/alginate mucoadhesive microspheres were assessed by Noreen et al. [25], as a potential sustained-release and mucoadhesive system to improve MTH bioavailability. However, very little work has been done on the microencapsulation of MTH using xanthan gum. A single study was conducted in 2016 by Ramalingam Nethaji et al. [26], where sodium alginate and different concentrations of natural mucoadhesive polymers such as xanthan gum and guar gum were used to develop gastroretentive formulations via the ionic gelation technique. In this study, the effect of the xanthan gum/guar gum mixture ratio on the properties of the microparticles obtained and on the release of MTH in the gastric medium (1.2) was evaluated. Optimal results were obtained with mixtures containing a higher fraction of xanthan. This contributed to the choice of polymer in this present study, alongside the other advantages offered by this polysaccharide.

In the literature, no study has been reported on the encapsulation of active substances into microparticles based exclusively on xanthan gum. In all the existing research, XG is often used either in the form of a mixture with other natural or synthetic polymers or else in the form of carboxymethyl xanthan [27,28,29,30,31]. Our present study is therefore a first.

The main objective of this work was to formulate an encapsulation system for metformin hydrochloride, based on xanthan gum, to control and modulate its release. The method used to develop this system was ionotropic gelation in the presence of aluminum chloride as a crosslinking agent. The microparticles obtained were then subjected to several physicochemical characterizations, such as particle size measurement, the swelling rate and the release kinetics in gastric and intestinal simulated media.

## 2. Materials and Methods

### 2.1. Materials

Metformin hydrochloride (MTH) was generously provided as a gift by Saidal Medea (Medea, Algeria); xanthan gum (XG), aluminium trichloride (AlCl_3_), sodium chloride (NaCl) and hydrochloric acid (HCl) were purchased from Sigma Aldrich (Hamburg, Germany). All other chemicals used were of analytical grade and were also purchased from Sigma Aldrich (Hamburg, Germany).

### 2.2. Fourier Transform Infrared (FTIR) Analysis

The compatibility study assessed by FTIR spectroscopy was conducted on the raw materials, namely MTH, XG and their mixture, and also on the formulated microspheres. The spectra with a resolution of 4 cm^−1^, using 10 scans, were then recorded in the range of 4000–500 cm^−1^ using a Fourier transform infrared spectrophotometer (Shimadzu, Tokyo, Japan).

### 2.3. Viscosity Measurement of Xanthan Gum Hydrogels

This test consisted of studying the variation in viscosity as a function of xanthan concentration. The rotational viscometer HAAKE Visco-Tester (VT5) was used for the viscosity measurements of xanthan gum solutions at the different concentrations of 0.5%, 0.75%, 1% and 1.25%. The tests were carried out at ambient temperature using an SP3 spindle at a shear rate of 60 rpm.

### 2.4. Formulation and Characterization of MTH Microparticles

Different solutions of xanthan gum (XG) were first prepared by the dispersion of a defined mass (0.5%, 0.75%, 1% and 1.25%) of this polymer in an aqueous solution of NaCl (0.1 M) under vigorous magnetic stirring (Table 1). The obtained solutions were left to rest overnight. Subsequently, the MTH was introduced into each xanthan gum solution and the mixture thus obtained was extruded dropwise using a 10 mL syringe into an AlCl_3_ solution at the concentration of 5% (*w*/*v*) with a rate of 0.5 mL/min. The capsules formed were left in the AlCl_3_ solution with magnetic stirring at 100 rpm for 1 h.

### 2.5. Particle Size Measurement

The particle size of the obtained microspheres was measured by a digital caliper having an accuracy of 0.001 mm. A random choice of microparticles for each analysis was made. A statistical analysis of the mean and the variation coefficient was carried out.

### 2.6. Swelling Study of MTH Microparticles

The swelling study was carried out in two dissolution media at pH 1.2 and pH 6.8 as a simulation of the gastric and intestinal medium, respectively. Approximately 50 mg of dry microparticles was carefully introduced into 25 mL of the dissolution medium. At regular time intervals, the particles were removed from the dissolution medium and stripped of excess liquid, before being weighed using a precision analytical balance. The swelling rates (SR) were calculated by the following equation [32]:

(1) $$\mathrm{SR}\;\left(\%\right)=\frac{Wg-Wi}{Wi}$$

where *Wi* and *Wg* are the mass of the microspheres in the initial state (at time t_0_) and in the swollen state (at time t), respectively.

### 2.7. Encapsulation Efficiency

The encapsulation efficiency (EE) values were calculated by an indirect assay of the amount of unencapsulated metformin hydrochloride (MTH) present in the filtrate recovered after filtration of the formulation of the capsules, at a wavelength of 234 nm. They were calculated by the following formula [9]:

(2) $$\;\mathrm{EE}\;\left(\%\right)=\frac{C_{n}-C_{0}}{C_{0}}$$

where C_0_ is the concentration of initial MTH and C_n_ is the concentration of unencapsulated MTH.

### 2.8. Calibration Curve

Before establishing the calibration curves, a scan in the UV–Vis domain (Perkin Elmer, Villebon-sur-yvette, France) was first carried out, in order to determine the optimum wavelength, λmax, corresponding to the absorption of the active principle metformin hydrochloride (MTH) in different media of dissolution. For the determination of λmax, 0.1 mg/mL solutions of metformin were prepared in buffers at pH 1.2 and pH 6.8. The results showed maximum absorption peaks defined at a wavelength λmax = 234 nm.

The calibration curves represent the variation in the absorbance (A) as a function of the solution’s concentration (A = f(c)). For metformin, the optical density measurements for solutions whose concentration varied between 0.006 mg/mL and 0.03 mg/mL were carried out in the two different buffer media, which were gastric buffer pH 1.2 and intestinal buffer pH 6.8 at 234 nm.

### 2.9. In Vitro Release Study

The in vitro dissolution study was carried out at two different pH values corresponding to simulated gastric media at pH 1.2 and intestinal media at pH 6.8 using a USP dissolution apparatus II, at the temperature of 37 ± 0.5 °C and the stirring speed of 100 rpm. An accurately weighted sample of microcapsules (100 mg) was introduced into the basket and placed in the dissolution medium. At defined time intervals [33], a 10 mL sample was withdrawn and filtered with a syringe filter (0.45 μm), and then replaced by the same volume with the dissolution medium. The samples obtained were then analyzed by UV–Vis (PerkinElmer Lambda 25) at the wavelength of 234 nm, after suitable dilution.

### 2.10. Statistical Analysis

The statistical analysis of all results was achieved via ANOVA using Tukey’s multiple comparison test. A *p*-value <0.05 was considered statistically significant. All experiments were carried out in triplicate.

## 3. Results and Discussion

### 3.1. Fourier Transform Infrared (FTIR) Analysis

FTIR spectroscopy gives information on chemical bonds and molecular structures but also allows the detection of the presence of intermolecular interactions in a mixture of compounds.

The FTIR spectrum of xanthan gum (XG) shows characteristic absorption bands at 1015.55 cm^−1^, 1407.66 cm^−1^, 1602.34 cm^−1^ and 1732.13 cm^−1^, corresponding, respectively, to the elongation of the ether function C–O–C, the C–H bonds of methyl groups and the asymmetric vibrations of COO–, as well as the elongation of CO esters (acetyls). The characteristic peak at 3317.00 cm^−1^ is attributed to the elongation of the OH hydroxyl groups [34,35,36,37].

The FT-IR spectrum of pure metformin hydrochloride shows two bands of valence vibration of the N–H bond of primary C–N–H lying in the region of 3400 cm^−1^ and 3100 cm^−1^. The absorbances at 3367 cm^−1^, 3290 cm^−1^ and 3149 cm^−1^ correspond to the symmetric and asymmetric elongation of primary N–H. An intense band positioned at 1621 cm^−1^ is assigned to the in-plane deformation of the NH_2_ bond. The bands at 735 cm^−1^ and 936 cm^−1^ of medium to low intensity are assigned to C–H and CN–C bond swings, respectively. Three deformation bands of the CH_3_ cluster of medium intensity are observed at 1413 cm^−1^, 1448 cm^−1^ and 1472 cm^−1^. Two characteristic bands located at 1038 cm^−1^ and 1166 cm^−1^ are attributed to the C–N valence vibration of a secondary amine. Two elongation vibrations of the C–Cl group are noted at 578 and 632 cm^−1^ [12,16].

**Figure 1:**
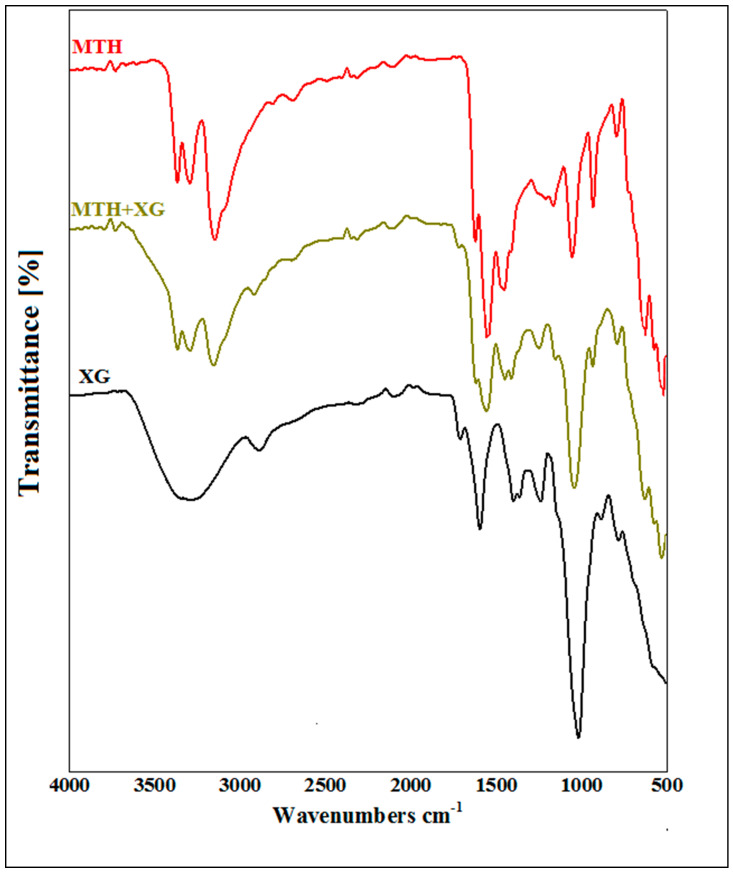
FTIR spectra of metformin hydrochloride (MTH), xanthan gum (XG) and their mixture (MTH+XG).

**Figure 2:**
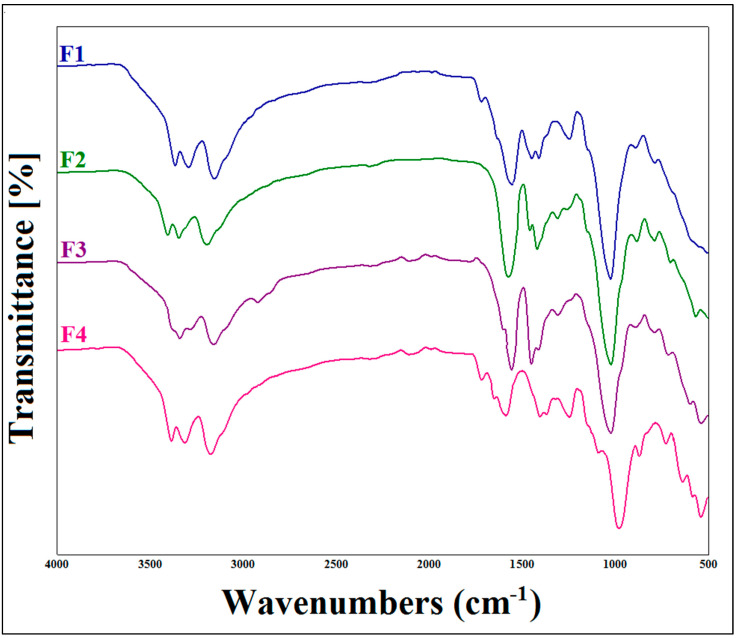
FTIR spectra of metformin hydrochloride (MTH) microparticles at different concentrations of xanthan gum.

The FTIR analysis of xanthan gum, metformin hydrochloride and their mixture confirmed the compatibility of MTH with XG. The appearance of the main characteristic peaks of MTH and XG on the spectrum of the mixture (MTH/XG) was noticed (Figure 1), with the observation of the N–H stretching of the primary amine group of metformin in the range of 3400 to 3100 cm^−1^, along with the presence of two bands at 1038 cm^−1^ and 1166 cm^−1^ [38] ascribed to C–N stretching, as well as the N–H deformation at 1602.34 cm^−1^ [12,16]. Characteristic absorption bands of XG were also observed at 1015.55 cm^−1^, 1407.66 cm^−1^ and 1732.13 cm^−1^, corresponding, respectively, to the elongation of the ether COC function, the CH bonds of the methyl groups [39] and the asymmetric vibrations of COO–, as well as the elongation of the acetyl group [34,35,36,37]. The FTIR spectra of the formulated microspheres, depicted in Figure 2, also demonstrate the absence of interactions between XG and MTH.

### 3.2. Viscosity Measurement of Xanthan Gum Hydrogels

The viscosity of a polymer solution defines its ability to resist flow, which essentially depends on the hydrodynamic volume of the polymer chains. Thus, it varies greatly depending on the average length of the chains and the polymer concentration, but also on the operating conditions (solvent, pH, temperature, ionic strength), which influence both the conformation and the flexibility of the chains [40].

**Figure 3:**
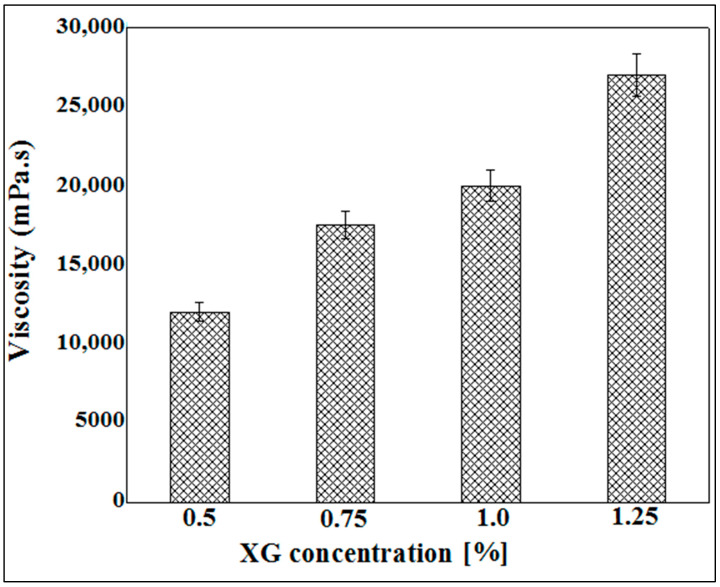
Viscosity measurement as a function of XG concentration varying between 0.5% and 1.25%.

Figure 3 represents the results of viscosity measurement as a function of the concentration of xanthan gum solution. The results obtained show that the apparent viscosity increases as a function of the concentration. Indeed, the more the polymer concentration increases, the more the polysaccharide chains intertwine, thus causing the greater entanglement of the network and consequently causing the rigidity of the hydrogel and its resistance to flow to become more pronounced.

These results led us to limit the use of xanthan gum to concentrations ranging from 0.5% to 1.25% (*w*/*v*). Indeed, at concentrations below 0.5%, the viscosity of the hydrogels obtained is very low, and, beyond 1.25%, the xanthan solution becomes highly viscous, and therefore obtaining capsules would be almost impossible. The extrusion of the xanthan gum solution through a syringe at concentrations greater than 1.25% is difficult to achieve and leads to the formation of misshapen microparticles.

### 3.3. Particle Size Measurement

**Figure 4:**
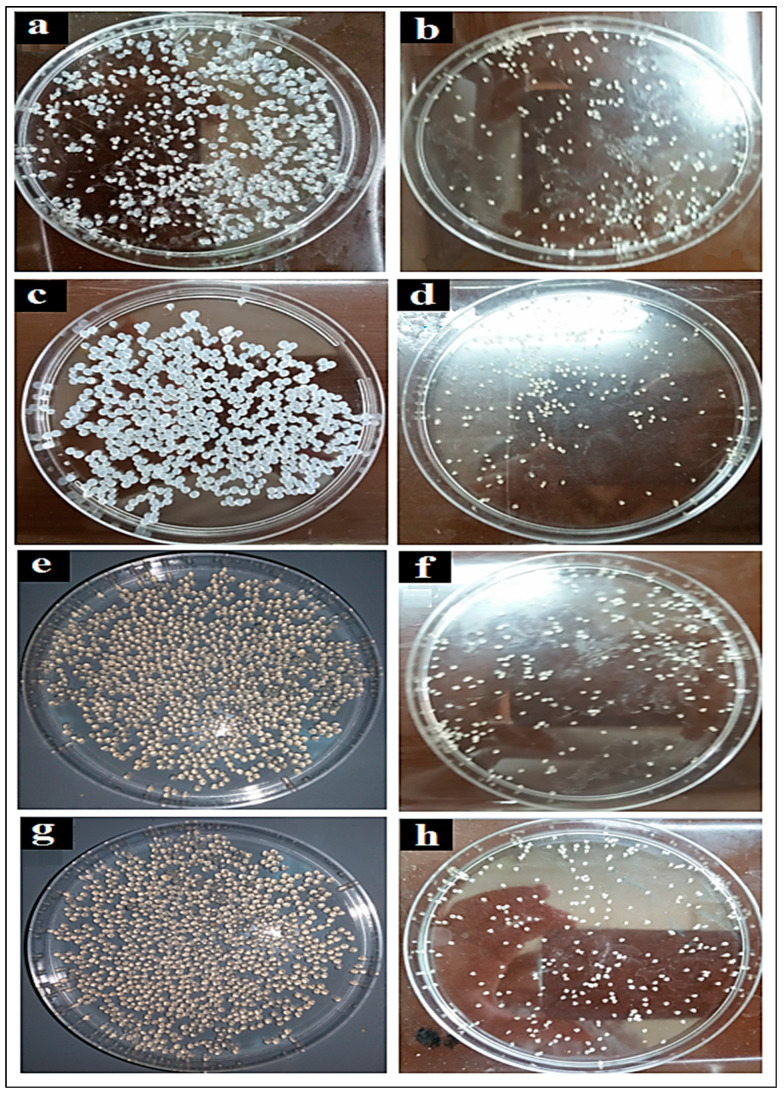
Shapes and appearance of the wet (**left**) and dry (**right**) microspheres: (**a**,**b**) at 0.5% (*w*/*v*), (**c**,**d**) at 0.75% (*w*/*v*), (**e**,**f**) at 1% (*w*/*v*), (**g**,**h**) at 1.25% (*w*/*v*).

The microspheres obtained (Figure 4) all had a homogeneous spherical shape, both in the wet and dry states.

**Figure 5:**
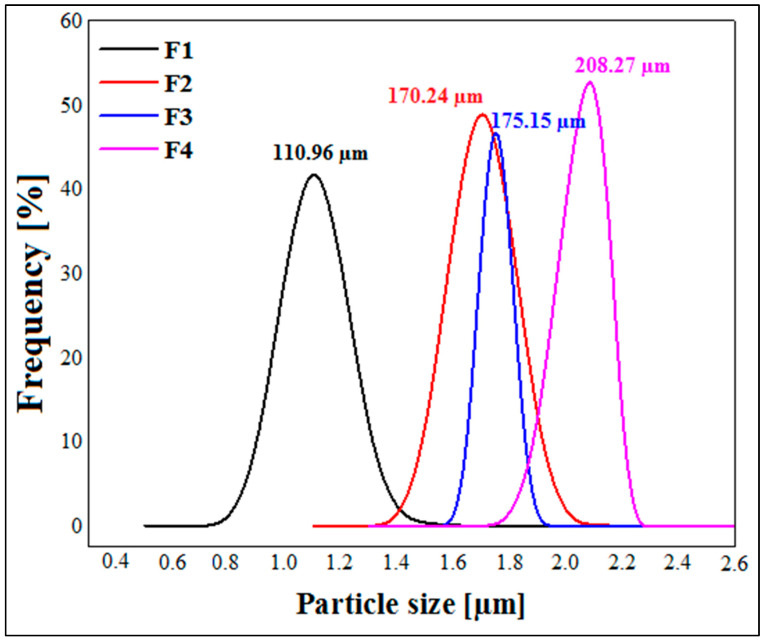
Particle size measurements of dried metformin hydrochloride microspheres.

Figure 5 illustrates the results of the particle size measurements. These results show that the size of the capsules varies from one formula to another depending on the concentration of xanthan gum. Indeed, the average diameter of the capsules for formula F1 is 110.96 ± 0.032 µm, and it increases with the increasing concentration of xanthan gum to finally reach a value of 208.27 ± 0.02 µm for formula F4, composed of 1.25% xanthan gum. This can be explained by the fact that the viscosity of the initial solution increases with the increase in the concentration of biopolymer; this leads to the formation of much larger capsules during extrusion through the syringe. Similar results were reported by Ramalingam Nethaji et al. [26] for metformin microspheres based on different ratios of sodium alginate, guar gum and xanthan gum. Kalpana et al. [41] also reported that the higher the concentration of chitosan, the higher the particle size of MTH microparticles. The results obtained by Hariyadi et al. [24] demonstrated the same tendency. All of these studies attributed the larger particle size with increasing concentrations of polymer to the greater viscosity of the media, which results in larger dimensions of the droplets formed.

### 3.4. Swelling Study

The swelling study based on gravimetry allows one to establish the kinetics of the penetration of the dissolution medium in the capsules. This study establishes its absorption rate and its increase in volume over time. This method consists of measuring the amount of liquid absorbed by the capsules as a function of time until equilibrium.

**Figure 6:**
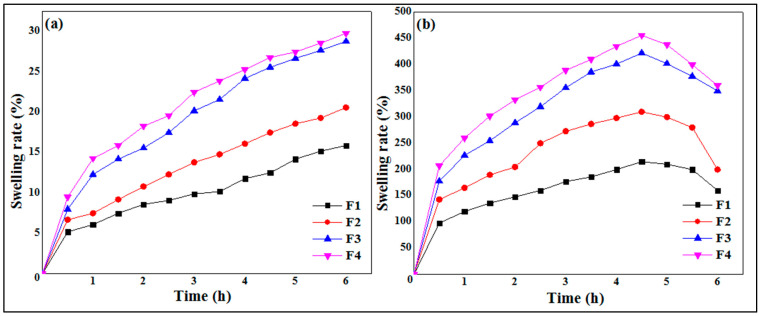
Swelling kinetics of MTH microspheres at pH 1.2 (**a**) and pH 6.8 (**b**).

The results of the swelling study are illustrated by Figure 6, where it is clearly shown that the swelling of the polymeric micro-sized spheres increases with time. Additionally, the swelling rate at pH 1.2 (Figure 6a) is much lower than at pH 6.8 (Figure 6b). At an acidic pH (pH slightly below 3.5), the neutralization of the charges leads to the bringing together of the chains, which insolubilize and precipitate. Below pH 3, the risk of chain hydrolysis is very high, which explains the low swelling rates in the pH 1.2 medium [42]. Xanthan gum is an anionic polysaccharide due to the presence of carboxylic functions –COOH in its structure. The slowing down of the swelling kinetics in an acid medium can be explained by the fact that the carboxylic groups are in the undissociated form (–COOH) at pH 1.2, therefore clearly reducing the electrostatic repulsion due to the neutralization of the charges and leading to the rapprochement of the chains. For pH values between 3.5 and 10, strong electrostatic repulsions between carboxylate groups tend to separate the chains from each other, producing viscous and stable solutions [42]. These data are in agreement with the high swelling rates obtained in pH 6.8 media and distilled water.

The results of the swelling study are depicted in Figure 6. It is observed that the swelling rate of the particles increases as the concentration of polymer concentration increases at pH 1.2 (Figure 6a) as well as at pH 6.8 (Figure 6b). The formula F1, based on 0.5% of XG, presented the lowest swelling rate against the formula F4, which showed a greater percentage and good swelling rate. These results are in total agreement with those found by Ramalingam Nethaji et al. [26], who attributed the increase in swelling index to the greater relative density in higher xanthan and guar gum concentrations and linked this to the presence of pores and cavities on the microspheres. Khonsari et al. [43] reported similar results for MTH microparticles based on Carbomer 934p and ethylcellulose and attributed the higher swelling to the fact that the liquid enters the particles through pores and binds to large particles, breaking the hydrogen bond.

### 3.5. Entrapment Efficiency

**Figure 7:**
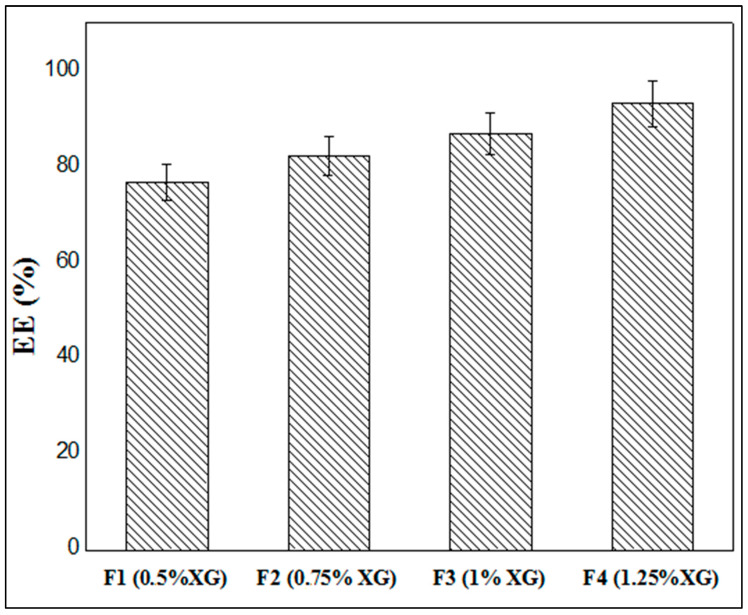
Entrappement efficiency of MTH microparticles.

Figure 7 illustrates the results of metformin entrapment efficiency (EE) as a function of XG concentration, where it is clearly shown that the rates of encapsulation of MTH vary between 76.75% and 93.11%, depending on the different formulas studied. It is also noted that the rate of encapsulation increases with the increase in the concentration of XG. Indeed, when the xanthan concentration is 0.5% (F1), the encapsulation rate is 76.75%; this rate reaches 82.27% when the xanthan gum concentration is 0.75% (F2) and passes to 86.94% in the formula F3 (1.0% XG), and finally reaches a maximum of 93.11% at XG concentrations of 1.25% (F4). These findings are in accordance with numerous literature references, including Ramalingam Nethaji et al. [26], Khonsari et al. [43] and Kalpana et al. [41].

This may be explained by the fact that the increase in the concentration of the biopolymer leads to the greater crosslinking of the latter by the Al^3+^ ions contained in the crosslinking solution. This causes the capsules to harden more quickly, preventing the active ingredient from emerging and being expelled to the outside environment. The low concentrations of xanthan gum lead to the formation of capsules whose membrane is much thinner, and therefore the presence of pores on the surface allows the early release of MTH and thus a lower rate of encapsulation.

### 3.6. Calibration Curve

**Figure 8:**
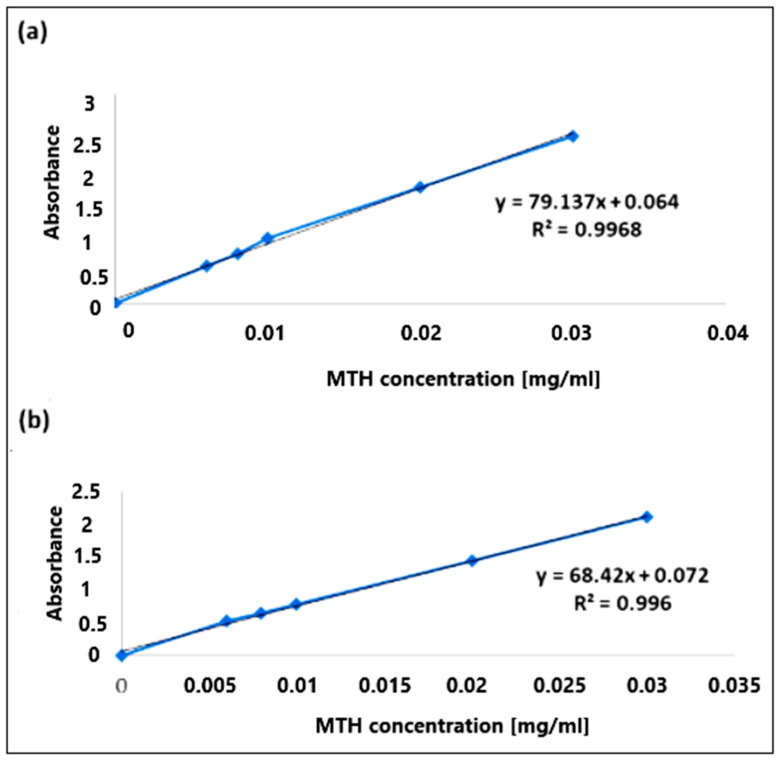
Calibration curves of metformin hydrochloride at pH 1.2 (**a**) and pH 6.8 (**b**).

The calibration curves were obtained by measuring the absorbance (A) at λ = 234 nm. The results obtained are shown in Figure 8. The calibration curves (A = f(c)) of metformin hydrochloride in simulated gastric and intestinal media were found to be linear in the concentration range of 0.006 mg/mL to 0.03 mg/mL, having a coefficient of regression value R^2^ = 0.997 at pH 1.2 (Figure 8a) and R^2^ = 0.996 at pH6.8 (Figure 8b).

### 3.7. In Vitro Release Study

**Figure 9:**
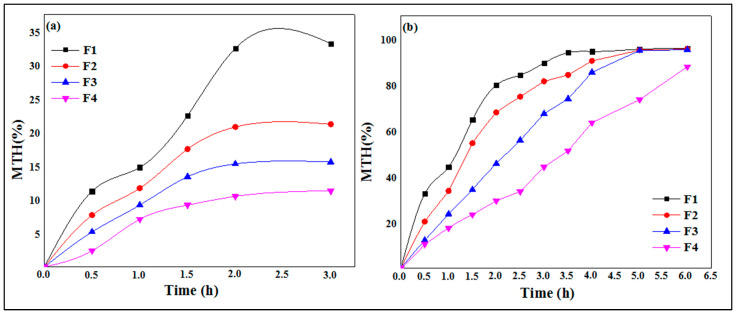
In vitro drug release of MTH from XG microspheres at pH 1.2 (**a**) and pH 6.8 (**b**).

The dissolution profiles show (Figure 9) an increase in the levels of MTH released as a function of time according to a non-linear relationship.

In the simulated gastric media pH 1.2 (Figure 9a), it is observed that the maximum release rates of MTH are greater in the F1 formulas (0.5 of XG %), with a value of 32.5% in 2.0 h, while this rate decreases when increasing the concentration of xanthan gum. Indeed, the release rates of metformin hydrochloride are, respectively, 20.8%, 15.3% and 10.5%, for F2 (0.75% of XG), F3 (1.0% of XG) and F4 (1.25% XG). These results indicate that with the increase in the biopolymer concentration, the hydrophilic matrix forming the capsules is more resistant to the release of metformin hydrochloride because of the entanglement of the polysaccharide chains, which becomes more and more important, hence reducing the porosity of the XG core.

The results of the in vitro release study of metformin hydrochloride as a function of time from the microcapsules of xanthan gum in the intestinal medium at pH 6.8 are shown in Figure 9b. These dissolution profiles show the gradual and continuous release of metformin over time. Formula F1, containing 0.5% XG, releases 90.2% of MTH in 3.0 h, while this rate decreases when increasing the concentration of xanthan gum, where the release rates of MTH are 90.4% after 4.0 h in formula F2 (0.75 XG%), 99.65% after 5.0 h in formula F3 (1.0% XG), 95.9% and finally a rate of 90.6% after 6.0 h in formula F4 (1.25% XG). At pH 6.8, the gel of the membrane is in a more hydrated state, increasing the permeability and the rate of MTH released. The xanthan gum is very hydrophilic; its network will swell and the aluminum ions will gradually diffuse in the external medium, which has the consequence of increasing the dimensions of the meshes of the network, and consequently the diffusion of MTH, until it degrades completely, because the polymer chains become too far from each other and the hydrogel layer disintegrates [44,45].

It is observed that the release rate of MTH is more prolonged in the acidic gastric simulated media compared to the intestinal simulated media. This may be due to the ionization of the xanthan gum, which is lower at an acidic pH due to the presence of –COOH groups onto the backbone of XG, thereby reducing the swelling of the polymeric network. The diffusion of the drug through the less swollen gelatinous polymer mass becomes more difficult, leading to more prolonged release. This result constitutes a promising practical approach for the development of gastroretentive delivery systems for the improvement of MTH bioavailability [29].

## 4. Conclusions

The encapsulation of metformin hydrochloride (MTH) in xanthan gum (XG) micrometric particles has been successfully carried out using the ionotropic gelation method. Initially, the interaction study between XG and MTH was conducted by Fourier transform infrared (FTIR) spectroscopy and, then, the xanthan gum hydrogels at different concentrations were submitted to viscosity testing.

The results of the FTIR analysis demonstrated the absence of interactions between MTH and XG, and the viscosity measurements led us to limit the use of xanthan gum to concentrations ranging from 05% to 1.25% (*w*/*v*). Afterward, the obtained particles were also characterized for their macroscopic appearance, particle size and encapsulation efficiency, in addition to the swelling and drug release studies. Particle size measurements confirmed the micrometric size of the xanthan particles, which varied between 110.96 ± 0.032 µm and 110.96 ± 0.032 µm. Furthermore, the encapsulation efficiency assessment demonstrated that the encapsulation rates of MTH varied between 76.75% and 93.0%, depending on the XG concentration, reaching a maximum in formula F4, which contained 1.25% of XG (F4). Low concentrations of xanthan gum led to the formation of microparticles with a much thinner membrane and therefore allowed the early release of MTH and a lower encapsulation rate. Additionally, this study showed that the swelling rate at pH 1.2 reached a maximum of 29.8%, while it was 360% at pH 6.8, at xanthan gum concentrations of 1.25% (F4). The release kinetics are also influenced upon increasing the biopolymer concentration. The kinetics of release of MTH were very low in the pH 1.2 medium, with higher rates for formula F1 (0.5% of XG), while this rate decreased when increasing the concentration of xanthan gum. At pH 6.8, the kinetics of release were more prolonged in time, with maximum release rates of 90.0% after 6 h found in formula F4 (1.25% GX). These results indicate that with an increasing biopolymer concentration, the hydrophilic matrix forming the xanthan gum capsules is more resistant to the release of metformin hydrochloride. The promising findings of this study demonstrate that it is worth exploring different operational conditions in future studies to optimize even further the amount of metformin hydrochloride encapsulated in the xanthan gum microparticles. Finally, it is concluded that the microparticles formulated with xanthan gum could be successfully used for the controlled release of drugs but also for the protection of pH-sensitive active ingredients, which could be degraded under the acidic conditions of the stomach. The potential blood-sugar-level-stabilizing effect of xanthan gum opens up prospects for the wider application of this macromolecule in the treatment and prevention of diabetes.

## Figures and Tables

**Table 1:** Composition matrix of the xanthan gum microparticles.

Ingredients	F1	F2	F3	F4
MTH (G)	1	1	1	1
XG (%)	0.5	0.75	1	1.25
ALCL3 (%)	5	5	5	5

## Data Availability

The data presented in this study are available in the manuscript.
